# Dark clouds of contrast

**DOI:** 10.1007/s12471-019-1257-y

**Published:** 2019-05-14

**Authors:** M. Boulaksil, J. E. M. Mellema, T. J. F. ten Cate

**Affiliations:** 1 Department of Cardiology, Radboud University Medical Center, Nijmegen, The Netherlands; 2 Department of Cardiology, Slingeland Hospital, Doetinchem, The Netherlands

A 59-year-old male patient was admitted to the Coronary Care Unit because of a non-ST-elevation acute coronary syndrome. An early angiography showed two-vessel coronary artery disease of a large obtuse marginal branch and the left anterior descending artery with two lesions (Fig. 1 and online video). What is your diagnosis?

**Fig. 1:**
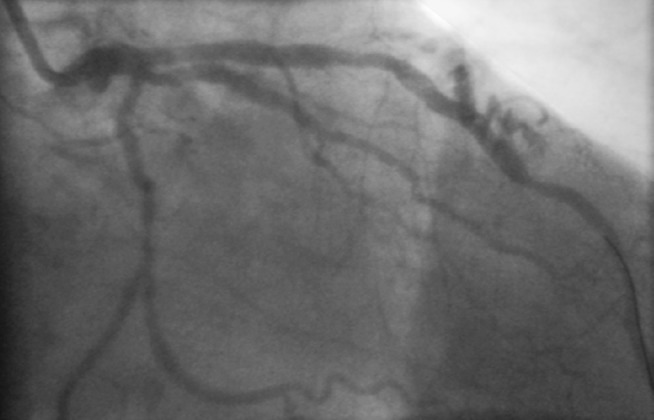
Still image of right anterior oblique (RAO) view of the coronary angiography of our patient (see also the online video (online supplement))

## Answer

You will find the answer elsewhere in this issue.

## Caption Electronic Supplementary Material

**Video 1. **Coronary angiography of our patient

